# Single-cell transcriptomic analysis of human pleura reveals stromal heterogeneity and informs *in vitro* models of mesothelioma

**DOI:** 10.1183/13993003.00143-2023

**Published:** 2024-01-25

**Authors:** Joanna Obacz, Jose Antonio Valer, Reshma Nibhani, Taylor S. Adams, Jonas C. Schupp, Niki Veale, Amanah Lewis-Wade, Jasper Flint, John Hogan, Giuseppe Aresu, Aman S. Coonar, Adam Peryt, Giulia Biffi, Naftali Kaminski, Hayley Francies, Doris M. Rassl, Mathew J. Garnett, Robert C. Rintoul, Stefan J. Marciniak

**Affiliations:** 1 Cambridge Institute for Medical Research (CIMR), University of Cambridge, Cambridge, UK; 2 Division of Respiratory Medicine, Department of Medicine, University of Cambridge, Cambridge, UK; 3 Section of Pulmonary, Critical Care, and Sleep Medicine, Yale School of Medicine, New Haven, CT, USA; 4 Department of Respiratory Medicine, Hannover Medical School, German Center for Lung Research (DZL), Hannover, Germany; 5 Royal Papworth Hospital NHS Foundation Trust, Cambridge, UK; 6 Cancer Research UK Cambridge Institute, University of Cambridge, Cambridge, UK; 7 Wellcome Sanger Institute, Wellcome Genome Campus, Hinxton, UK; 8 Joint first authors; 9 Joint senior authors

## Abstract

The pleural lining of the thorax regulates local immunity, inflammation and repair. A variety of conditions, both benign and malignant, including pleural mesothelioma, can affect this tissue. A lack of knowledge concerning the mesothelial and stromal cells comprising the pleura has hampered the development of targeted therapies. Here, we present the first comprehensive single-cell transcriptomic atlas of the human parietal pleura and demonstrate its utility in elucidating pleural biology. We confirm the presence of known universal fibroblasts and describe novel, potentially pleural-specific, fibroblast subtypes. We also present transcriptomic characterisation of multiple *in vitro* models of benign and malignant mesothelial cells, and characterise these through comparison with *in vivo* transcriptomic data. While bulk pleural transcriptomes have been reported previously, this is the first study to provide resolution at the single-cell level. We expect our pleural cell atlas will prove invaluable to those studying pleural biology and disease. It has already enabled us to shed light on the transdifferentiation of mesothelial cells, allowing us to develop a simple method for prolonging mesothelial cell differentiation *in vitro*.

## Tweetable abstract

**The first single-cell RNA-seq atlas of human parietal pleura is now accessible *via* a free web-based data-mining tool, MesothelialCellAtlas.com, and illuminates aspects of benign and malignant pleural biology** https://bit.ly/3u3eDu8

## Introduction

The pleura regulates local immunity and wound-healing [1]. Perturbing these functions induces fibrosis; in the case of exposure to asbestos, chronic irritation can cause pleural mesothelioma, a rapidly progressive incurable cancer [1, 2]. The lack of tractable *in vitro* models represents a significant barrier to the study of pleural biology. Although protocols for mesothelial cell extraction have been published [3, 4], *in vitro* culture is plagued by rapid transdifferentiation to fibroblast-like cells, the precise nature of which is unclear.

Epithelioid mesothelioma is the most differentiated primary pleural cancer and has a median survival of only 18 months, while sarcomatoid mesothelioma is typically fatal in under a year [5]. Although mesothelioma has a relatively modest mutational burden, breast cancer type 1 susceptibility protein-associated protein 1 (*BAP1*) and merlin (*NF2*) mutations are detected in 49% and 44% of cases, respectively [6]. Mesothelioma is a stromal-rich malignancy, but which cells are reprogrammed or recruited to become mesothelioma cancer-associated fibroblasts (CAF) is unknown. While the heterogeneity of tissue-specific fibroblasts has been studied in other tissues, the fibroblasts of the pleura remain mysterious. However, in some circumstances, mesothelial cells can transdifferentiate to CAFs, demonstrated by the fact that the peritoneal mesothelium contributes CAFs to pancreatic cancer and peritoneal metastasis [7, 8].

Tissue fibroblasts have traditionally been defined by their spindle shape, their expression of canonical markers such as thymocyte differentiation antigen 1 (*THY1*) and platelet-derived growth factor receptor-α (*PDGFRA*), and as the source of extracellular matrix (ECM) [9]. Recently, it has become apparent that considerable diversity exists among fibroblasts, with some possessing tissue-specific characteristics [10, 11]. Analysis of single-cell transcriptomes suggests that both universal and tissue-specific subtypes of fibroblasts exist [11].

To address the paucity of information available about the stromal cells of the pleura, we generated a single-cell transcriptome atlas of healthy adult human parietal pleura, elucidating its cellular composition and phenotypic diversity. In doing so, we identified universal and novel populations of fibroblasts. Transcriptomic and functional studies demonstrated a key role for transforming growth factor-β (TGF-β) in mesothelial-to-mesenchymal transition (MMT). Finally, by using the atlas to validate the transcriptomes of two-dimensional (2D) mesothelial cell cultures, we were able to compare gene expression in healthy mesothelium with a panel of early passage mesothelioma 2D models, revealing phenotypic groups.

## Materials and methods

### Subjects and samples

Patients undergoing video-assisted thoracoscopic surgery for pneumothorax were enrolled with Royal Papworth Hospital Research Tissue Bank Generic Research Ethics Committee approval (table 1). All patients provided written informed consent and samples were linked anonymised.

**TABLE 1:** Clinical characteristics of donors

**Sample**	**Age (years)**	**Sex**	**Comorbidities**	**Duration since most recent pneumothorax (days)**	**Total number of ipsilateral pneumothoraces**
**S1**	27	F	Autoimmune hepatitis	81	1
**S2** ^#^	17	F	–	25	2
**S3**	29	M	Asthma	300	1
**S4**	40	M	–	104	2
**S5**	50	M	–	15	1
**S6**	34	M	–	4380	2
**S7**	16	M	–	11	1
**S8**	28	M	–	30	3
					
**N1**	37	M	–	4	1
**N2**	35	F	Asthma	8	1
**N3**	29	F	AD PCKD	122	2
**N4** ^#^	17	F	–	25	2
**N5**	54	M	Sarcoidosis Emphysema	8	1

S: single-cell RNA-sequencing parietal pleura sample; N: bulk RNA-sequencing primary mesothelial cell sample; F: female; M: male; AD PCKD: autosomal dominant polycystic kidney disease. ^#^: donor S2 and N4 are the same individual.

### Single-cell RNA-sequencing

For single-cell RNA-sequencing (scRNA-seq), pleural specimens were immersed in cold RPMI supplemented with antibiotics and typically processed within 1 h. Pleura was washed three times in PBS, minced with scalpels and digested in RPMI supplemented with collagenase I (5 mg·mL^−1^, Life Technologies) and DNase I (100 µg·mL^−1^, Sigma) for 2.5 h at 37°C. Digested tissue was filtered through a 100-μm cell strainer, washed with PBS and centrifuged at 800 *g*. Cell pellets were resuspended first in red blood cell lysis buffer then in RPMI (2% fetal bovine serum (FBS)). Dead cells and cellular debris were removed by sorting the 7-amino actinomycin D (7-AAD)-negative population with BD FACSMelody Cell Sorter (BD Biosciences). A total of 16 000 cells per patient were submitted to 10x Genomics and immediately loaded on 10×3′ chip. scRNA-seq libraries were generated using the 10x Chromium platform 3ʹ v3.1 kit. Library quality was assessed following cDNA synthesis and after completion of the 3′ Gene Expression Libraries on the Agilent TapeStation system employing DNA High Sensitivity D5000 and D1000 chips, respectively (all Agilent Technologies). Single-cell libraries were sequenced on an Illumina NovaSeq 6000 instrument using 28 bp length sequencing for read 1 and 90 bp length sequencing for read 2, and aiming for 20 000 reads per cell. Raw reads were trimmed with Cutadapt (v1.17) [12] for read 2, 5′ anchored template switch oligo contamination and read 2, 3′ polyA sequence contamination. Trimmed, paired reads with <30 bp of remaining read 2 were discarded. Trimmed reads were then aligned with the STARsolo [13] implementation of STAR (v2.7.6a) to human genome GRCh38.p13 with Gencode annotation release 38 (www.gencodegenes.org/human/release_38.html). Cell barcodes from the “filtered” STARsolo outputs were aggregated in R (www.r-project.org). Cells with >20% of transcripts from mitochondrial origin were discarded. Downstream cell-type annotation analysis was conducted using the package Seurat (v4.0.2) (https://satijalab.org/seurat). The data are available at the Gene Expression Omnibus repository with accession number GSE243446.

The Seurat package was applied in an iterative process of dimension reduction, graph-embedding, clustering, cell-type annotation and subsetting of similar cell types that was performed recursively until we were satisfied that all heterotypic multiplets were removed. Highly variable genes were generated and used to perform principal component (PC) analysis. Significant PCs were determined using JackStraw analysis and visualisation of heatmaps focusing on PCs 1 to 20. PCs 1 to 10 were used for graph-based clustering (at res=0.8) for all samples. These groups were projected onto a Uniform Manifold Approximation and Projection (UMAP) analysis run using previously computed PCs 1 to 10. The cluster-specific marker genes were identified by running the FindAllMarkers function in Seurat to the normalised gene expression data. Cell-type identities were validated based on expert curation of the distinctiveness and stability of a phenotype across sample replicates; labels were assigned based on concordance with the literature. We used clusterProfiler from Bioconductor (v4.4.4) (https://bioconductor.org) to perform biological processes enrichment analyses and pySCENIC (v0.11.2) (https://scenic.aertslab.org) for gene regulatory network interference. Count data transformation was performed using the variance stabilising transformation implemented in the Bioconductor DESeq2 package (v1.36.0). For each gene, the dispersion was calculated to measure its variance among samples, and thus the 1000 genes with highest dispersions were selected for clustering analysis. Hierarchical clustering analysis was performed using the Pearson correlation coefficient as the distance metric and Ward's linkage rule. The pySCENIC package (v0.11.2) with two gene-motif rankings (hg38__refseq-r80__10kb_up_and_down_tss and hg38__refseq-r80__500bp_up_and_100bp_down_tss) from the RcisTarget database was used to investigate the gene regulatory network of transcription factors [14]. Areas under the recovery curve were calculated with a threshold set at 0.35. Bulk RNA-seq and scRNA-seq data were integrated using single-cell identification of subpopulations with bulk sample phenotype correlation (Scissor) analysis [15].

To validate clustering analysis, we performed an alternative dimensionality reduction in Scanpy (v1.9.3) using PaCMAP (release 0.7.0) (https://github.com/YingfanWang/PaCMAP) [16]. For the pleural cell populations, PaCMAP was used with default parameters. Clustering was performed using Leiden clustering at a 0.07 resolution with 100 nearest neighbours and Gauss method. For the stromal population, PaCMAP was used with further pairs ratio parameter equal to 1 and other parameters set as default. Clustering was performed using Leiden clustering at a 0.06 resolution with 100 nearest neighbours and Gauss method. 2D plots were coloured by PaCMAP obtained clusters, or by the previous UMAP clustering to observe cell clustering between methods. Sankey plots were obtained using the pySankey package (https://github.com/anazalea/pySankey).

### Bulk RNA-sequencing

For sequencing analysis, paired-end transcriptome reads were quality filtered and mapped to GRCh38 (ensemble build 98) using STAR-v2.5.0c [17] with a standard set of parameters (https://github.com/cancerit/cgpRna). Resulting bam files were processed to get per gene read count data using HTSeq 0.7.2 [18] with parameters --stranded=reverse and --mode=union. We calculated transcripts per million values using the count and transcript length data for further downstream analysis. Mutations in *BAP1* and *NF2* were obtained from Cell Model Passports (CMP) and the cell line genomic datasets are available from the CMP database (https://cellmodelpassports.sanger.ac.uk). RNA-seq data are available *via* CMP: study ID EGAS00001005728, dataset IDs EGAD00001009642 and EGAD00001010924.

### Mesothelial cell isolation and culture

For preferential isolation of mesothelial cells, parietal pleura was finely chopped and incubated in 0.25% trypsin/0.01% EDTA at 37°C for 30 mins. Detached cells were collected by centrifugation at 800 *g* and red blood cells removed by incubating the pellet with red blood cell lysis buffer (Life Technologies). After washing with PBS, mesothelial cells were pelleted and resuspended in 50:50 RPMI (with 10% FBS, penicillin/streptomycin, 2 µg·mL^−1^ heparin and 1 µg·mL^−1^ hydrocortisone) and BEBM (with BEGM SingleQuot Suppl&Growth Factors, Lonza). Every 2–3 days media were refreshed until a confluent monolayer was obtained (typically 5–10 days). Mesothelioma cell lines were obtained from Mesobank UK [19] and cultured as previously described [20]. To detect MMT markers of primary mesothelial cells, TGF-β inhibitors SB431542 and Noggin were added to the media at 10 µM and 100 ng·mL^−1^, respectively, until reaching confluence.

For purity validation, mesothelial cells were isolated as above and cultured for 3 days in 50:50 RPMI:BEBM media. Adherent cells were detached with TrypLE (Gibco) and incubated in 2% bovine serum albumin (BSA) solution for 30 mins at 4°C, then with unconjugated primary antibodies for 30 mins at 4°C, then washed. Fluorophore-conjugated secondary antibodies were then added for a further 30 mins at 4°C. Populations were sorted using a BD Influx Cell Sorter (BD Biosciences). Debris was excluded through DAPI staining. Sorted populations were re-plated, passaged then re-assayed by flow cytometry.

### Flow cytometry

For flow cytometry analysis, single-cell suspensions were obtained by enzymatic digestion then incubated with saturating concentrations of immunoglobulins for 20 min at room temperature. Specific cell populations were detected with primary antibodies directly conjugated with fluorescent marker (table 2) for 30 min at 4°C. Populations were analysed or sorted using a BD LSRFortessa Cell Analyzer and BD Influx Cell Sorter, respectively (BD Biosciences). Dead cells were excluded using 7-AAD staining. Data were analysed using FlowJo (BD Biosciences).

**TABLE 2:** Antibodies used in the study

**Target**	**Visualisation**	**Dilution**	**Manufacturer**
**CD45**	Brilliant Violet 421 anti-human	1:40	BioLegend (368521)
**CD31**	Brilliant Violet 421 anti-human	1:40	BioLegend (303123)
**CD140b (PDGFRβ)**	PE anti-human	1:20	BioLegend (323605)
**CD146 (MUC18)**	Alexa Fluor 488 anti-human	1:20	BioLegend (361019)
**COL15A1**	Immunohistochemistry	1:100	Atlas (HPA017915)
**D2-40 (PDPN)**	Immunohistochemistry	Prediluted	CellMarque (760-4395)
**PDPN**	APC/Cyanine7 anti-human	1:100	BioLegend (337029)
**PI16**	Immunohistochemistry	1:100	Abbexa (abx102279)
**PI16**	Immunohistochemistry	1:100	Abbexa (abx173977)
**HLA**	–	1:50	Abcam (ab96569)
**COL15A1**	–	1:200	Novus Biologicals (nbp1-91088)
**MSLN**	–	1:50	Abcam (ab196235)
**CD74**	–	1:100	Abcam (ab108393)
**HLA-DR**	–	1:100	Abcam (ab92511)
**HLA-DP**	–	1:100	Abcam (ab227675)
**Mouse IgG**	Alexa Fluor 647 anti-human	1:500	Life Technologies (A11008)
**Rabbit IgG**	Alexa Fluor 647 anti-human	1:500	Life Technologies (A31573)
**Rabbit IgG**	Alexa Fluor 488 anti-human	1:500	Life Technologies (A21240)

### Quantitative PCR

Total RNA was extracted using Qiagen RNeasy Kit for quantitative real-time PCR (qPCR), reverse transcribed with MultiScribe Reverse Transcriptase (ThermoFisher Scientific) and analysed using the CFX96 Touch Real-Time PCR Detection System (Bio-Rad) and SYBR Green JumpStart *Taq* ReadyMix (Sigma). Samples were normalised to actin gene using 2^ΔΔ^CT-method and analysed by one-way ANOVA with Tukey's multiple comparisons test. Primers used are listed in table 3.

**TABLE 3:** Primers used in the study

**Gene**	**Forward primer**	**Reverse primer**
***ACT***	5′-GCACCACACCTTCTACAATGA-3′	5′- GTCATCTTCTCGCGGTTGGC-3′
***ITLN1***	5′-AATGGACCTGTTCTTCGT-3′	5′-TCTGGGTAGACTGCTTTG-3′
***LUM***	5′-TCATCCCTGGTTGAGCTGGAT-3′	5′-AGGATAATGGCCCCAGGATCT-3′
***MFAP5***	5′-GTGACTCAAGCGACTCCAGAA-3′	5′-AGTCATCTGTGGAAGGTGCAAT-3′
***MSLN***	5′-CTATTCCTCAACCCAGATGCGT-3′	5′-GCACATCAGCCTCGCTCA-3′
***PDGFRA***	5′- TGCGGGTGGACTCTGATAATGC-3′	5′- GTGGAACTACTGGAACCTGTCTCG-3′
***UPK3B***	5′-TGTGTCTTCGATGGGCTTGCCA-3′	5′-AATGTCAGCCAGTGTCTCCGGGTT-3′

### ELISA

For ELISA, mesothelial cells were cultured in 50:50 RPMI (with 10% FBS, penicillin/streptomycin, 2 µg·mL^−1^ heparin and 1 µg·mL^−1^ hydrocortisone) and BEBM (with BEGM SingleQuot Suppl&Growth Factors). When cells reached 80% confluency, media was replaced with a fresh 1 mL. After 48 h, passage 0 medium was collected and cells were passaged with fresh medium. At passage 1, 10 μM of SB431542 (TGF-β type 1 receptor inhibitor) was added. Medium was collected for passages 1 and 2 as above. TGF-β1 concentration was measured using a human TGF-β1 ELISA kit (ab100647; Abcam) following the manufacturer's instructions.

### Electron microscopy

For electron microscopy, cells were grown on Melinex plastic coverslips (TAAB Laboratory Equipment Ltd), rinsed in 0.9% saline and fixed in 2% glutaraldehyde/2% formaldehyde in 0.05 M sodium cacodylate buffer containing 2 mM calcium chloride at 4°C. After washing, samples were transferred to liquid nitrogen-cooled brass inserts and freeze-dried overnight, mounted on aluminium scanning electron microscope stubs and coated with 25 nm gold then viewed using a FEI/Thermo Fisher Scientific Verios 460 scanning electron microscope run at 2.0 kV and 50 pA probe current. For immunohistochemistry of formalin-fixed, paraffin-embedded tissue sections, antigen retrieval was done by heating slides in 10 mM citric acid buffer (pH 6.0) for 15 min. All sections were counterstained with Gill's haematoxylin and imaged with a ZEISS Axio Observer.

### Immunofluorescence microscopy

Formalin-fixed, paraffin-embedded tissue sections were deparaffinised and rehydrated. Heat-induced antigen retrieval was performed using a 10 mM sodium citrate buffer with 0.05% Tween-20 (pH 6.0) at 95°C for 20 min. Sections were blocked using blocking buffer (PBS with 1% BSA and 0.1% Tween-20) for 1 h and incubated overnight at 4°C in a wet chamber with the primary antibody (table 2). Sections were then incubated with a fluorophore-conjugated secondary antibody at room temperature for 1 h before applying Vector TrueVIEW Autofluorescence Quenching Kit Working Solution to minimise background fluorescence, and mounted with VECTASHIELD Vibrance with DAPI Antifade Mounting Medium (Vector Laboratories, Inc.). Slides were imaged using the ZEISS LSM 880 Confocal Microscope with Airyscan and processed using ZEN (blue edition) software (ZEISS).

### Immunohistochemistry

Formalin-fixed, paraffin-embedded tissue sections were deparaffinised and rehydrated. Heat-induced antigen retrieval was performed using a 10 mM sodium citrate buffer with 0.05% Tween-20 (pH 6.0) at 95°C for 15–20 min. Hydrogen peroxidase block was performed for 15 min followed by further incubation with blocking buffer for 1 h. Abcam protein block was applied for 10 min and then sections were incubated in primary antibody at 4°C overnight in a wet chamber. Sections were incubated with biotinylated goat anti-polyvalent for 10 min and covered with streptavidin peroxidase for a further 10 min. 3, 3′-diaminobenzidine (DAB) chromogen (HRP/DAB (ABC) Detection Kit, Abcam) was added to DAB substrate, applied to sections, and incubated for 1–3 min. Haematoxylin counterstain was performed, washed in tap water and slides dipped into Scott's Tap Water Substitute. Sections were dehydrated and mounted using DPX (Fischer Scientific). Slides were imaged with an Axio Imager optical microscope (ZEISS).

## Results

### Mapping stromal heterogeneity in the human pleura

**FIGURE 1:**
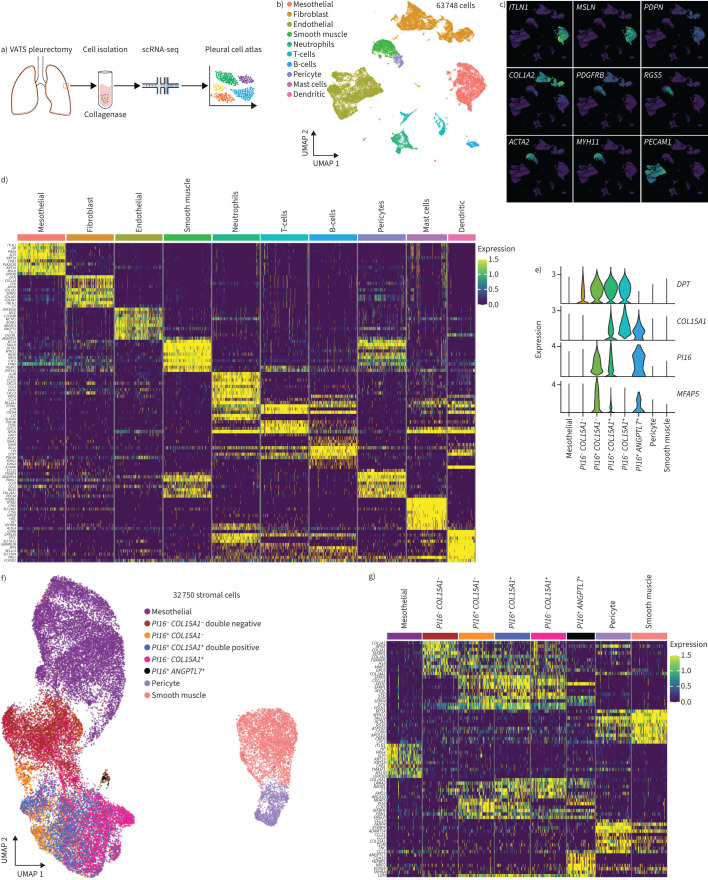
A single-cell RNA-sequencing (scRNA-seq) atlas of human parietal pleura. a) Study pipeline: samples obtained by video-assisted thoracic surgery (VATS) were disaggregated to single cells with collagenase. Viable cells were collected by fluorescence-activated cell sorting and immediately processed by 10x Genomics Chromium Controller. b) Uniform Manifold Approximation and Projection (UMAP) of 63 748 pleural cells from eight donors are colour-coded by cell type. c) UMAP projection of canonical marker gene expression: mesothelial cells (intelectin-1 (*ITLN1*), mesothelin (*MSLN*) and podoplanin (*PDPN*)); fibroblasts (collagen type I ɑ2 chain (*COL1A2*) and platelet-derived growth factor receptor β (*PDGFRB*)); pericytes (regulator of G protein signaling 5 (*RGS5*)); smooth muscle cells (smooth muscle α-actin-2 (*ACTA2*) and myosin heavy chain 11 (*MYH11*)); and endothelial cells (platelet and endothelial cell adhesion molecule 1 (*PECAM1*)). d) Heat map of the relative average expression of the most strongly enriched genes for each cluster (presented as log(fold change) of one cluster *versus* all others), grouped by cell type. All gene expression values are normalised across rows. e) Violin plots of dermatopontin (*DPT*), collagen type XV ɑ1 chain (*COL15A1*), peptidase inhibitor 16 (*PI16*) and microfibril-associated protein 5 (*MFAP5*) expression. f) UMAP visualisation of 32 750 stromal cells. Eight populations identified through graph-based clustering are indicated by colour and annotated as mesothelial, *PI16*^−^ *COL15A1*^−^, *PI16*^+^ *COL15A1*^−^, *PI16*^+^ *COL15A1*^+^, *PI16*^−^ *COL15A1*^+^, *PI16*^+^ *ANGPTL7*^+^, pericyte and smooth muscle. g) Heat map of the relative average expression of the most strongly enriched genes for each cluster in f, presented as log(fold change) of one cluster *versus* all others, grouped by cell type. All gene expression values are normalised across rows. *ANGPTL7*: angiopoietin-related protein 7.

Parietal pleura was donated by eight adults undergoing video-assisted thoracoscopic surgery pleurectomy for spontaneous pneumothorax (table 1, figure 1a). The median age was 28.5 years (range 16–50 years); six participants were male. scRNA-seq was performed on freshly isolated pleural cells using a droplet-based approach and data were combined to generate a pleural single-cell atlas of 63 748 cells (figure 1b). The results can be explored through the Mesothelial Cell Atlas data mining portal (MesothelialCellAtlas.com). Ten clusters were identified, in most cases representing all participants (figure 1b, supplementary figure S1). Based on canonical markers, clusters were found corresponding to stromal, endothelial, immune and mesothelial cells (figure 1c and d, supplementary figure S2). To characterise stromal and mesothelial components, we selected clusters expressing mesothelin (*MSLN*), *PDGFRA*, platelet-derived growth factor receptor-β (*PDGFRB*), lumican (*LUM*), smooth muscle α-actin-2 (*ACTA2*) and myosin heavy chain 11 (*MYH11*) and identified 32 750 cells. Signature genes were cross-referenced to known markers to identify subclusters corresponding to mesothelial (n=11 535), smooth muscle (n=4245), pericyte (n=1194) and at least five populations of fibroblast cells (figure 1e–g, supplementary figure S3).

In most tissues, dermatopontin-positive (*DPT^+^*) “universal fibroblasts” comprise subtypes marked by expression of peptidase inhibitor 16 (*PI16*) or collagen type XV ɑ1 chain (*COL15A1*) [10, 11]. To unpick the heterogeneity of fibroblasts in parietal pleura, we examined the expression of these markers and observed *DPT^+^*fibroblasts that were also positive for either or both *PI16* and *COL15A1* (figure 1e and f). *PI16* expression has been reported for fibroblasts in vascular niches [11], while microfibril-associated protein 5 (*MFAP5*) is detected in vascular adventitial pulmonary fibroblasts [21]. In keeping with a vascular adventitial phenotype, *MFAP5* was detected in many of the pleural *PI16*^+^ *COL15A1*^−^ fibroblasts (n=1864, figure 1e–g). *PI16*^−^ *COL15A1*^+^ cells have been proposed to represent universal parenchymal fibroblasts secreting basement membranes [11]. We found these cells were abundant in human parietal pleura (n=4787, figure 1e–g). We detected many pleural fibroblasts with an intermediate double-positive phenotype (n=4410)*,* expressing genes characteristic of both *PI16*^+^ *COL15A1*^−^ fibroblasts (cellular communication network factor 5 (*CCN5*), insulin-like growth factor binding protein 6 (*IGFBP6*), fibrillin 1 (*FBN1*) and proline and arginine rich end leucine rich repeat protein (*PRELP*)) and *PI16*^−^ *COL15A1*^+^ fibroblasts (laminin subunit ɑ2 (*LAMA2*), neuronal growth regulator 1 (*NEGR1*), complement C7 (*C7*) and ATP-binding cassette subfamily A member 10 (*ABCA10*)), although unlike *PI16*^+^ *COL15A1*^−^ cells, they expressed relatively low levels of MFAP5 (figure 1e–g, supplementary figure S4). In addition, a rare subpopulation of *PI16^+^* fibroblasts (n=80 cells, 0.2% of stromal cells) was detected, which selectively expressed angiopoietin-related protein 7 (*ANGPTL7*), a gene implicated in regulating stemness in resting hair-follicle stem cells (figure 1e–g, supplementary figure S5) [22, 23], and additional genes associated with stemness (supplementary figure S7). When clustering was repeated using a separate method (PaCMAP), similar results were obtained, attesting to the robustness of these populations (supplementary figure S6).

### Distinct functional subsets of fibroblasts are detected in benign human pleura

**FIGURE 2:**
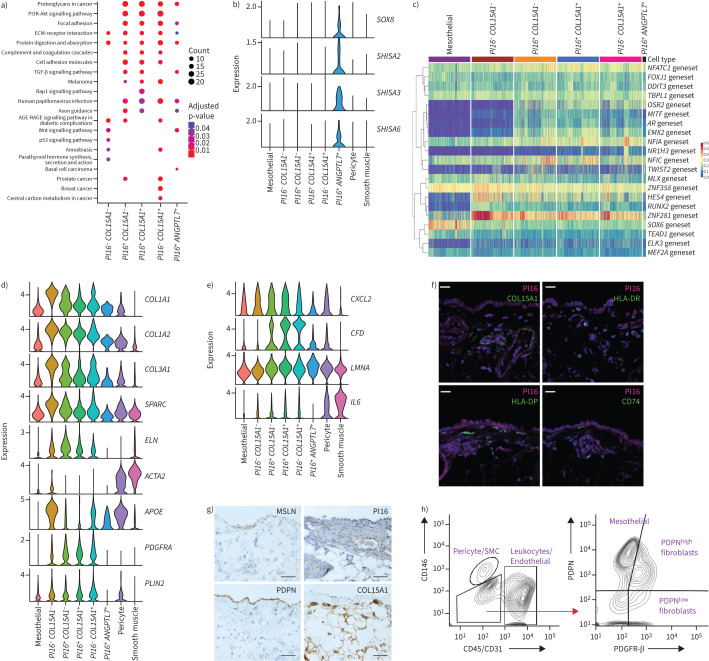
Stromal heterogeneity in healthy pleura. a) Kyoto Encyclopedia of Genes and Genomes pathway gene set enrichment analysis of pleural fibroblasts. ECM: extracellular matrix. b) Violin plots of the expression of SRY-box transcription factor 8 (*SOX8*) and Shisa family members *SHISA2*, *SHISA3* and *SHISA6*. c) Heat map of gene regulatory network analysis. d) Violin plots of the expression of collagen type I ɑ1 chain (*COL1A1*), collagen type I ɑ2 chain (*COL1A2*), collagen type III ɑ1 chain (*COL3A1*), secreted protein acidic and cysteine rich (*SPARC*), elastin (*ELN*), actin ɑ2, smooth muscle (*ACTA2*), apolipoprotein E (*APOE*), platelet-derived growth factor receptor-α (*PDGFRA*) and perilipin 2 (*PLIN2*). e) Violin plots of the expression of C-X-C motif chemokine ligand 2 (*CXCL2*), complement factor D (*CFD*), lamin A/C (*LMNA*) and interleukin 6 (*IL6*). f) Co-immunofluorescence micrograph of human parietal pleura stain for peptidase inhibitor 16 (PI16) (magenta) and collagen type XV ɑ1 chain (COL15A1) (green, top left), human leukocyte antigen-DR (HLA-DR) (green, top right), human leukocyte antigen-DP (HLA-DP) (green, bottom left) and CD74 (green, bottom right). Scale bars: 20 µm. g) Representative immunohistochemically stained images of mesothelin (MSLN), podoplanin (PDPN), COL15A1 and PI16 in human pleura tissue sections. Scale bars: 50 µm. h) Fluorescence-activated cell sorting of pleural stromal components. SMC: smooth muscle cell; PDGFR-β: platelet-derived growth factor receptor-β.

Gene set enrichment analysis of stromal populations was performed to gain functional insight. In *PI16^+^* fibroblast clusters, pathway analysis of differentially expressed genes identified patterns associated with focal adhesion, ECM-receptor interaction, PI3K-Akt signalling and TGF-β signalling (figure 2a). Similar pathways were detected in *PI16*^−^ *COL15A1*^+^ fibroblasts, though TGF-β signalling was less prominent. A substantial population of fibroblasts failed to express either *PI16* or *COL15A1* (n=4635, figure 1f). These double-negative cells lacked evidence of PI3K-Akt and TGF-β signalling, but expressed genes associated with ECM interaction (figure 2a). The *ANGPTL7*^+^ subpopulation of *PI16*^+^ fibroblasts showed evidence of WNT and TGF-β signalling, with ECM-related functions being less evident (figure 2a). This suggests a progenitor-like role, as has been suggested for other *PI16*^+^ fibroblasts [11], so it is noteworthy that other stemness genes (including SRY-box transcription factor 8 (*SOX8*) and Shisha family members (*SHISA*)) were selectively expressed in this population (figure 2b, supplementary figure S7). A similar population of cells named endoneurial fibroblasts has previously been described in peripheral nerves [24].

Gene regulatory network analysis was performed to infer activity of transcription factor regulons across stromal populations. Mesothelial cells displayed greater SRY-box transcription factor 6 (SOX6) regulon activity than pleural fibroblasts (figure 2c). Consistent with this, our scRNA-seq atlas confirmed SOX6 was expressed more robustly in mesothelial cells than stromal components (supplementary figure S8). This is consistent with the previous detection of SOX6 in mesothelioma cells in the pleura, peritoneum and tunica vaginalis [25–27].

Double-negative *PI16*^−^ *COL15A1*^−^ fibroblasts showed activation of the regulons of Hes family BHLH transcription factor 4 (HES4), a Notch-responsive transcription factor implicated in regulating stromal cell specialisation [28], and zinc finger protein 281 (ZNF281), an EMT-inducing transcription factor (figure 2c) [29, 30]. The transcription factors HES4 and, to a lesser extent, ZNF281 were expressed in a proportion of the *PI16*^−^ *COL15A1*^−^ population (supplementary figure S8). Double-negative cells strongly expressed components of the collagen-rich ECM (collagen type I ɑ1 chain (*COL1A1*), collagen type I ɑ2 chain (*COL1A2*), collagen type III ɑ1 chain (*COL3A1*) and secreted protein acidic and cysteine rich (*SPARC*)), although other canonical myofibroblast markers (elastin (*ELN*), *ACTA2*, transgelin (*TAGLN*) and *MYH11*) were detectable only at low levels (figure 2d, supplementary figure S9). Double-negative cells also expressed apolipoprotein E (*APOE*), a marker of lipofibroblasts, but low levels of other lipofibroblast genes (*PDGFRA* and perilipin 2 (*PLIN2*)) (figure 2d).

Fibroblasts regulate local immunity [10, 31]. We therefore searched for evidence of immune modulatory fibroblasts in benign pleura. *DPT*^+^ marks both universal fibroblasts and inflammatory fibroblasts (figure 1) [10, 11, 31], and additional inflammatory fibroblast-associated genes (C-X-C motif chemokine ligand 12 (*CXCL12*), complement factor D (*CFD*) and lamin A/C (*LMNA*)) were detected in some *PI16^+^* *COL15A1*^+^ double-positive fibroblasts, while other canonical inflammatory CAF (iCAF)-like marker genes (interleukin 6 (*IL6*), angiotensin II receptor type 1 (*AGTR1*), hyaluronan synthase 1 and 2 (*HAS1* and *HAS2*)) were less expressed (figure 2e, supplementary figure S10a). In benign pleura, we detected low levels of human leukocyte antigen-DR-ɑ (HLA-DRA), human leukocyte antigen-DP-ɑ1 (HLA-DPA1) and human leukocyte antigen-DR-β1 (HLA-DRB1) expression in some universal fibroblasts (supplementary figure S10b). The expression of *CD74*, which encodes the invariant major histocompatibility complex (MHC) class II γ chain, suggests that functional MHC class II may be synthesised. Immunofluorescence microscopy demonstrated the presence of a small number of PI16^+^ cells expressing HLA-DP, HLA-DR or CD74 (figure 2f).

Immunofluorescence also detected cells either single- or double-positive for PI16 and/or COL15A1 (figure 2f). Immunohistochemical analysis confirmed MSLN and podoplanin (PDPN) expression by the mesothelium (figure 2g). To confirm the presence of the stromal cell populations detected by scRNA-seq, we used a flow cytometry panel comprising PDPN, PDGFR-β and CD146 to distinguish mesothelial cells, fibroblasts, pericytes and smooth muscle. Immune cells and endothelial cells were depleted based on CD45 and CD31 expression respectively, while pericytes and smooth muscles were identified by CD146. CD45^−^ CD31^−^ CD146^−^ triple-negative cells could be further separated into PDPN^+^ PDGFR-β^−^ mesothelial cells, PDPN^low^ universal and PDPN^high^ fibroblasts (figure 2h).

These data demonstrate that benign human pleura comprises multiple fibroblast populations including known *DPT^+^* universal subtypes. An abundant pleural *PI16*^−^ *COL15A1*^−^ double-negative population was found to display features of lipo- and myofibroblasts, and may serve as a source of fibrillar collagens in the parietal pleura. A low abundance population of *PI16*^+^ *ANGPTL7*^+^ cells expressed genes suggesting a progenitor-like behaviour. Some *DPT*^+^ fibroblasts expressed genes involved in immune modulation, while certain mesothelial cells and *DPT*^+^ fibroblasts expressed MHC class II, suggesting a potential immunomodulatory role in benign pleura.

### TFG-β drives MMT

**FIGURE 3:**
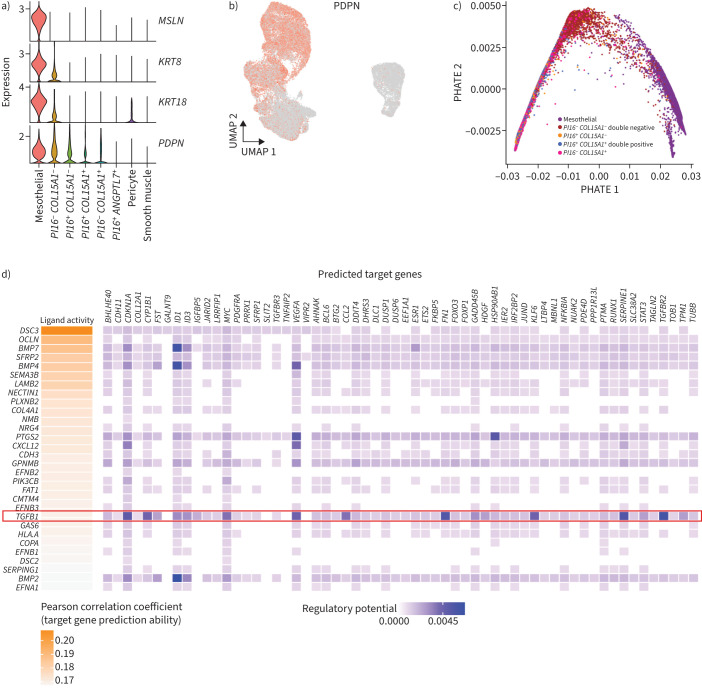
Isolation and dedifferentiation of parietal pleural mesothelial cells. a) Violin plots of the expression of mesothelin (*MSLN*), keratin 8 (*KRT8*), keratin 18 (*KRT18*) and podoplanin (*PDPN*). b) Uniform Manifold Approximation and Projection (UMAP) visualisation of PDPN expression in mesothelial and stromal cells. c) Potential of Heat Diffusion for Affinity-based Trajectory Embedding (PHATE) visualisation of pseudotime trajectory of mesothelial transdifferentiation into activated fibroblasts. d) Intracellular communication between mesothelial cells and activated fibroblasts as determined by NicheNet algorithm. Ligand activity prediction showing 20 mesothelial ligands with the highest likelihood (Pearson correlation coefficient) of affecting the gene expression in the receiver-activated fibroblasts. Ligands specifically interacting with activated fibroblasts (but not universal fibroblasts) are depicted in red. Ligand-target matrix denoting the regulatory potential between mesothelial ligands and target genes in activated fibroblast. *PI16*: peptidase inhibitor 16; *COL15A1*: collagen type XV ɑ1 chain.

Our single-cell data revealed that several genes highly expressed in mesothelial cells (keratin 8 (*KRT8*), keratin 18 (*KRT18*), *PDPN* and *MSLN*) were expressed at lower levels in *PI16*^−^ *COL15A1*^−^ double-negative fibroblasts (figure 3a and b). Little is known about MMT in non-cancerous pleura or the cellular fate of transitioning mesothelial cells. To address this, we performed pseudotime analysis using Potential of Heat Diffusion for Affinity-based Trajectory Embedding (PHATE) [32], which suggested that mesothelial cells transition preferentially to *PI16^−^* *COL15A1^−^* fibroblasts (figure 3c). To understand the drivers of pleural MMT, we modelled ligand-receptor interactions using NicheNet to identify ligands potentially promoting fibroblast growth [33]. Ligand activities were ranked and Pearson coefficients were calculated for the correlation between targets of a given ligand and the expression of genes in the receiver cell (figure 3d). With mesothelial cells as senders and double-negative fibroblasts as receivers, NicheNet nominated TGF-β1 as a putative ligand signalling from mesothelial to *PI16*^−^ *COL15A1*^−^ pleural fibroblasts.

**FIGURE 4:**
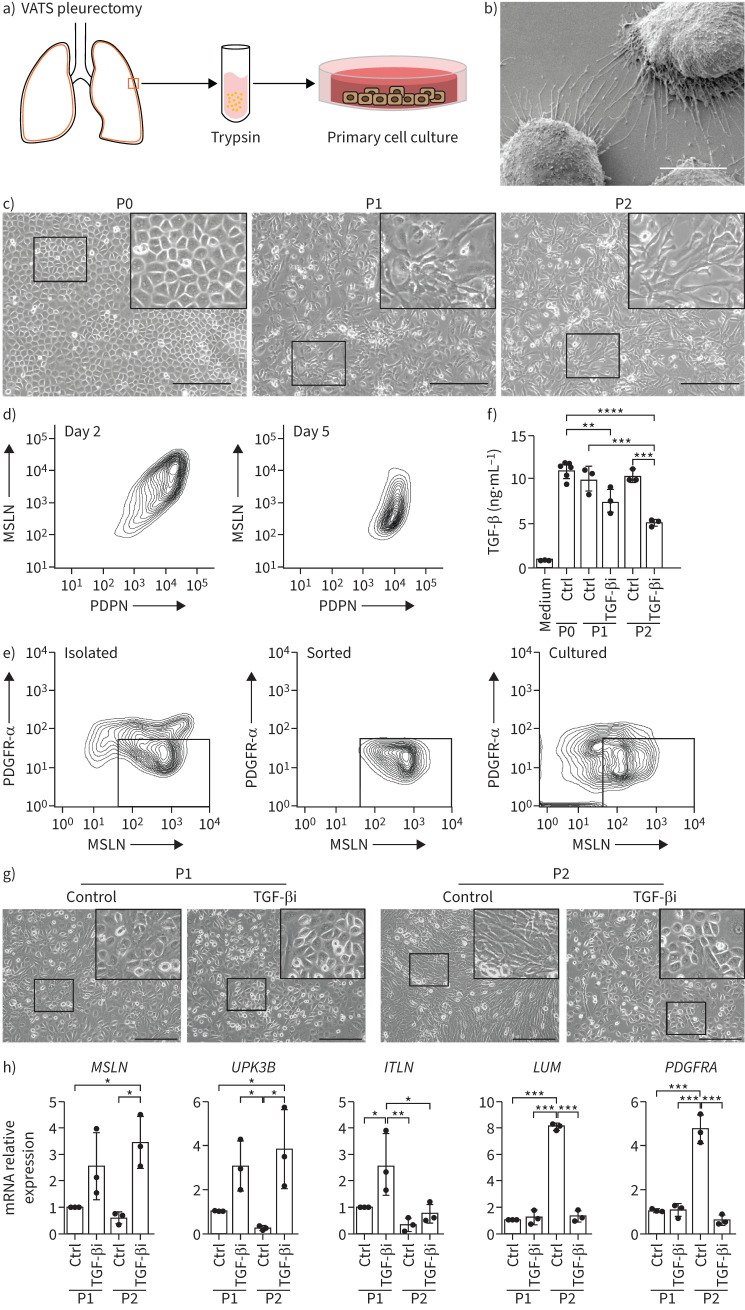
Transforming growth factor-β (TGF-β) signalling regulates pleural mesothelial-to-mesenchymal transition. a) Mesothelial cells isolation pipeline: samples obtained by video-assisted thoracic surgery (VATS) were subjected to limited trypsinisation protocol. The detached surface mesothelial cells were cultured on plastic substratum for 5–6 days post-surgery. b) Scanning electron micrograph of low confluence trypsin-isolated mesothelial cells cultured for 5 days post-surgery. Scale bar: 20 µm. c) Phase contrast images of primary mesothelial cell cultures at passage 0, day 5 post-surgery (P0); passage 1, day 7 post-surgery (P1); and passage 2, day 14 post-surgery (P2). Scale bars: 300 µm. Insert shows zoomed image of boxed region. d) Fluorescence-activated cell sorting (FACS) of trypsin-isolated mesothelial cell cultures at day 2 and day 5, stained for mesothelin (MSLN) and podoplanin (PDPN). e) FACS of trypsin-isolated mesothelial cell cultures stained for platelet-derived growth factor receptor-ɑ (PDGFR-α) and MSLN. Cells were gated for PDGFR-α^low^ and MSLN^high^. These were plated and cultured until P2. f) TGF-β ligand concentrations in fresh medium (Medium) or conditioned medium from primary mesothelial cell at P0, P1 and P2, without (Ctrl) or with TGF-β inhibitor (TGF-βi) SB431542 (10 µM). g) Phase contrast images of primary mesothelial cell cultures at P1 (day 5) and P2 (day 7), without (Control) or with TGF-βi SB431542 (10 µM) and TGF-βi Noggin (100 ng·mL^−1^). Scale bars: 300 μm. Insert shows zoomed image of boxed region. h) Mesothelial and fibroblast gene expression without (Ctrl) or with TGF-β inhibition by quantitative real-time PCR. Three independent experiments are presented (dots for each experiment and histogram for mean). *UPK3B*: uroplakin 3B; *ITLN1*: intelectin-1; *LUM*: lumican. *: p<0.05; **: p<0.01; ***: p<0.001; one-way ANOVA with Tukey's multiple comparisons test.

To examine MMT *in vitro*, we isolated primary mesothelial cells from pleura by limited trypsinisation, which unlike collagenase treatment mobilises few fibroblasts (figure 4a). These mesothelial cells formed monolayers with a cobblestone morphology and expressed MSLN and PDPN (table 1, figure 4b–d). Electron microscopy confirmed the presence of microvilli typical of mesothelial cells (figure 4b). Subsequently, during *in vitro* culture, primary mesothelial cells changed morphology, acquiring an elongated, spindle shape resembling fibroblasts (figure 4c). In parallel, MSLN staining weakened, though PDPN staining was retained for at least 5 days (figure 4d). To gain confidence that this reflected MMT rather than overgrowth of a contaminating population of fibroblasts, we stained P0 cells with antibodies to fibroblast marker PDGFR-α and mesothelial marker MSLN (figure 4e). A majority of cells were MSLN^high^, consistent with the previously observed mesothelial morphology. Most were PDGFR-α^low^, though some MSLN^+^ cells also expressed PDGFR-α. We gated for PDGFR-α^low^ MSLN^high^ cells and cultured these to P2. Again, MSLN staining fell. Many cells acquired PDGFR-α positivity, supporting MMT.

To test whether TGF-β sustains MMT *in vitro*, we cultured mesothelial cells from at least three donors in the presence or absence of the TGF-β type I receptor inhibitor SB431542. Little TGF-β was present in fresh medium, while media from mesothelial cell cultures contained 10 ng·mL^−1^ TGF-β, which was reduced by treatment with TGF-β inhibitor (figure 4f). As expected, in the absence of TGF-β inhibition, mesothelial cells started to lose their cobblestone morphology on passaging (figure 4g). *In vitro* culture of trypsin-isolated cells was associated with a reduction of mesothelial marker gene expression (*MSLN*, uroplakin 3B (*UPK3B*) and intelectin-1 (*ITLN1*)) and an increase in fibroblast gene expression (*LUM* and *PDGFRA*) (figure 4h). By contrast, cultures treated with the TGF-β inhibitors retained mesothelial cell morphology and marker gene expression without increased fibroblast gene expression. This suggests that TGF-β, derived locally from mesothelial cells, may drive transdifferentiation of mesothelial cells to fibroblasts *in vitro*.

### Linking scRNA-seq atlas with 2D models of the mesothelium and mesothelioma

**FIGURE 5:**
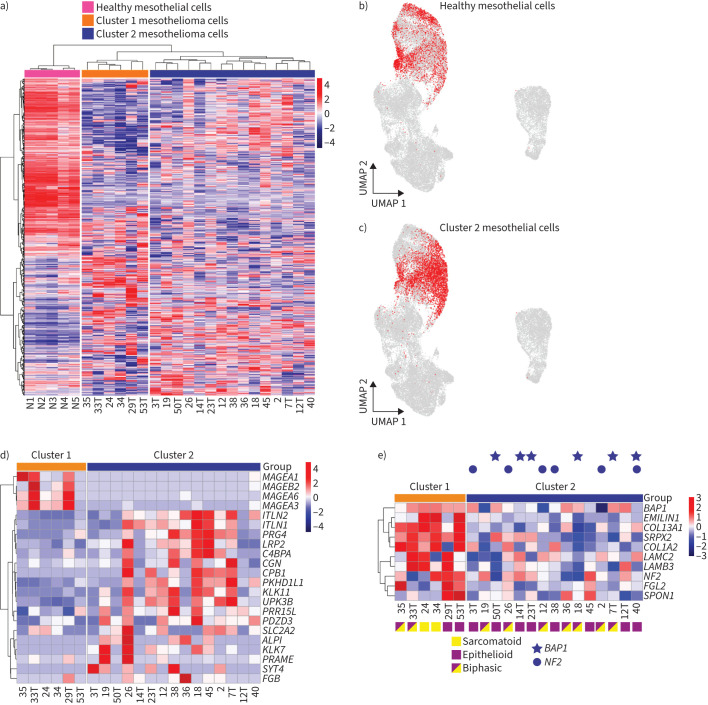
Characterisation of two-dimensional models of mesothelioma. a) Unsupervised classification of gene expression data (bulk RNA-sequencing (RNA-seq)) of five primary mesothelial cell cultures cultured for 5–6 days post-surgery (N1–N5) and 21 mesothelioma cell lines. Count data transformation was performed by using the variance stabilising transformation implemented in the DESeq2 package. For each gene, the dispersion was calculated to measure its variance among samples, and thus the 1000 genes with highest dispersions were selected for clustering analysis. Hierarchical clustering analysis was performed using the Pearson correlation coefficient as the distance metric and Ward's linkage rule. b) Single-cell identification of subpopulations with bulk sample phenotype correlation (Scissor) analysis to identify stromal cells in the single-cell RNA-seq (scRNA-seq) atlas with similar gene expression to healthy mesothelial cell cultures. c) Scissor analysis to identify stromal cells in the scRNA-seq atlas with similar gene expression to cluster 2 mesothelioma cell lines. d) Unsupervised classification of gene expression data (bulk RNA-seq) of 21 mesothelioma cell lines divided into clusters 1 and 2 as defined in a. Hierarchical clustering analysis was performed using the Pearson correlation coefficient as the distance metric and Ward's linkage rule. Genes were selected from those most differentially expressed in epithelioid *versus* sarcomatoid mesothelioma [34]. e) Heat map of “ECM structural constituents” gene set (breast cancer type 1 susceptibility protein-associated protein 1 (BAP1), elastin microfibril interfacer 1 (*EMILIN1*), collagen type XIII ɑ1 chain (COL13A1), sushi repeat containing protein X-linked 2 (SRPX2), collagen type I ɑ2 chain (*COL1A2*), laminin subunit γ2 (LAMC2), laminin subunit β3 (LAMB3), merlin (NF2), fibrinogen-like 2 (*FGL2*) and spondin 1 (*SPON1*)) in cluster 1 compared to cluster 2. *BAP1* mutations indicated by star. *NF2* mutation indicated by circle. Cell lines derived from sarcomatoid tumour=yellow square, from epithelioid=purple square, from biphasic tumour=purple/yellow square. UMAP: Uniform Manifold Approximation and Projection.

Analysis of *in vitro* models of mesothelioma is limited by a paucity of authentic benign controls. To determine if our primary mesothelial cells recapitulate their counterparts *in vivo*, we used bulk RNA-seq to examine the transcriptomes of early cultures (5–6 days post-isolation) from five individuals. In parallel, we performed bulk RNA-seq of a panel of 21 early passage mesothelioma cell lines (passage 5–8) [19, 20]. The 1000 most differentially expressed genes were selected for hierarchical clustering (figure 5a). All primary benign cultures (N1–N5) clustered together, as did the mesothelioma lines. To compare the bulk RNA-seq data with our scRNA-seq atlas of parietal pleura we used Scissor analysis [15]. This confirmed that our primary mesothelial cultures mapped well to cells in the mesothelial scRNA-seq cluster (figure 5b).

Having generated a valid control dataset, we compared this with the transcriptomes of early passage mesothelioma. By Gene Ontology (GO) term analysis, mesothelioma cells exhibited higher expression of genes associated with growth factor activity and ECM production, but lower immune receptor activity (supplementary figure S11). By Kyoto Encyclopedia of Genes and Genomes (KEGG) pathway analysis, mesothelioma cells showed less gene expression associated with inflammation (supplementary figure S11). Within the 21 mesothelioma cell lines, two clusters were observed. When these clusters were examined by Scissor (figure 5c), cluster 2 showed similarity to mesothelial cells *in vivo*, while cluster 1 was dissimilar to all healthy pleural cell populations. Cluster 2 was enriched for genes expressed preferentially in epithelioid mesotheliomas compared with sarcomatoid disease: *ITLN1*, *ITLN2*, proteoglycan 4 (*PRG4*), LDL receptor related protein 2 (*LRP2*), complement component 4 binding protein-ɑ (*C4BPA*), cingulin (*CGN*), carboxypeptidase B1 (*CPB1*), PKHD-like 1 (*PKHD1L1*), kallikrein-related peptidase 11 (*KLK11*) and *UPK3B* (figure 5d) [34]. Conversely, several genes reported to be expressed in sarcomatoid mesothelioma were preferentially expressed in cluster 1: members of the MAGE family *MAGEA1*, *MAGEA3*, *MAGEB2* and *MAGEA6*.

Comparing cluster 1 with cluster 2 by GO term analysis, we observed higher ECM structural constituents in cluster 1 consistent with a sarcomatoid phenotype (figure 5e). Genomic sequencing revealed that *BAP1* and *NF2* pathogenic variants, which are more common in epithelioid tumours [2], were detected only in cluster 2. Of the 21 early passage mesothelioma cultures examined, two had originated from individuals with proven sarcomatoid mesothelioma; both of these were in cluster 1.

These data suggest that early passage parietal mesothelial cells isolated by limited trypsinisation efficiently model benign mesothelial cells *in vivo*. This provides an important control for disease states, while TGF-β inhibition may allow healthy mesothelial cultures to be maintained *in vitro* for longer to facilitate such work. Our comparison highlights differences in mesothelioma cell line gene expression that might influence their local environment, including the ECM and the immune system. We also found that early passage mesothelioma cell lines adopt two main phenotypes sharing similarity with epithelioid and sarcomatoid mesothelioma, but these do not always reflect the diagnostic label of the donor.

## Discussion

We analysed 63 748 cells by scRNA-seq from eight individuals to define the cellular components of human parietal pleura. In doing so, we identified known and previously undescribed fibroblast subtypes. Comparison of the *in vivo* transcriptomes of parietal mesothelium with those of mesothelial cells grown in 2D culture validated our protocol for mesothelial cell isolation, while comparisons with early passage mesothelioma cells revealed two phenotypic groups, which may have value in identifying subtype-specific therapies.

Although the physiological functions of human pleura have been described [1], the interactions between pleural cell components have yet to be studied in detail. We set out to generate a parietal pleural cell atlas and in doing so uncovered functional heterogeneity of parietal pleural fibroblasts. We identified *COL15A1*^+^ and *PI16*^+^ universal fibroblasts corresponding to parenchymal and adventitial populations, and a novel *COL15A1*^−^ *PI16^−^* subtype abundant in the parietal pleura. This subtype displayed a phenotype related to lipofibroblasts, *e.g.* APOE expression, while showing myofibroblast features, including expression of collagen-rich ECM components suggesting a role as the pleural source of fibrillar collagens. We detected a putative rare population of *PI16*^+^ *ANGPTL7^+^* fibroblasts, whose transcriptomic signature suggests a progenitor-like function. Similar cells named endoneurial fibroblasts have been described in peripheral nerves and so these may derive from innervation of the pleura [24]. It is important to note, however, that without eventual validation these remain a putative cell type.

A number of previously described lung fibroblast subtypes, including alveolar and peribronchial subtypes, appear to be absent from the parietal pleura (supplementary figure S10c) [35–37]. By contrast, the *PI16*^+^ universal adventitial fibroblasts we detected shared characteristics of murine and human lung adventitial fibroblasts (*e.g.* expression of *MFAP5*, scavenger receptor class A member 5 (*SCARA5*) and *PI16*) [35, 36, 38]. Subpleural lung fibroblasts were recently described [35, 36, 37] and share some characteristics with the *PI16*^−^ *COL151A1*^−^ putative parietal pleural fibroblasts we report (*e.g.* myristoylated alanine rich protein kinase c substrate-like 1 (*MARCKSL1*)). However, differences exist, *e.g.* relatively few parietal pleural fibroblasts express the subpleural fibroblast markers apolipoprotein C1 (*APOC1*) or matrix metallopeptidase 23B (*MMP23B*). Intriguingly, the expression of *HAS1* in *PI16*^−^ *COL15A1*^−^ double-negative fibroblasts of healthy parietal pleural is shared by subpleural fibroblasts from the lungs of patients with idiopathic pulmonary fibrosis, but not those from healthy lung [37].

Mesothelial cells are versatile and under appropriate conditions transdifferentiate into adipocytes, osteoblasts [39], myofibroblasts [40] and even vascular smooth muscle [41]. Here, using pseudotime modelling and *in vitro* assays, we support the multipotent nature of pleural mesothelial cells showing transdifferentiation to fibroblasts. We demonstrate that antagonising TGF-β delays transdifferentiation of mesothelial cells in culture, consistent with similar roles for TGF-β signalling at other sites such as the peritoneum [42]. Conversely, the networks maintaining mesothelial differentiation remain unclear, though we observed SOX6 regulon activity to be higher in mesothelial cells than other pleural components, and SOX6 itself to be detectable predominantly in mesothelial cells. Double-negative fibroblasts showed expression of HES4 and activation of its regulon, both of which were absent in mesothelial cells. Given that HES4 is a Notch-responsive transcription factor implicated in stromal cell specialisation [28], further work should clarify its role in the pleura.

We observed that some *PI16*^+^ *COL15A1*^+^ double-positive pleural fibroblasts appear to express genes associated with inflammatory fibroblasts, although additional cues may be necessary to manifest a full iCAF-like phenotype, given that other canonical iCAF marker genes were not expressed. Antigen-presenting CAFs are another type of immune-modulating fibroblast that express MHC class II and modulate CD4^+^ T-cells [7, 31] and it has been suggested that peritoneal mesothelial cells may be progenitors of antigen-presenting CAFs in pancreatic cancer [7]. In this regard it is intriguing that HLA-DRA, HLA-DPA1 and HLA-DRB1 expression was observed in some universal fibroblasts. While the detection of CD74 and other MHC class II proteins by immunofluorescence microscopy in a small number of PI16^+^ cells supports these scRNA-seq data, we acknowledge that ambient RNA contamination is a potential confounder and so future studies focused on this small subset of cells will be required.

In the present work, we provide a simple protocol for growing benign pleural mesothelial cells whose transcriptomes mirror those of mesothelial cells *in vivo*. To showcase the utility of our pleural cell atlas, we explored the transcriptome of primary low passage mesothelioma cells. Unsupervised clustering based on their transcriptomes yielded two groups: one sharing similarity with healthy mesothelial cells *in vivo*, and a second dissimilar to any healthy pleural cell type. Comparison with published tumour RNA-seq data suggests that cluster 2 may serve as a model for epithelioid mesothelioma, while cluster 1 more closely resembles sarcomatoid mesothelioma. This information may help guide subtype-targeted drug discovery.

In summary, we have generated a single-cell atlas of human parietal pleura revealing heterogeneity among its stromal components and identifying novel fibroblast subtypes. We showed utility of these data by identifying and manipulating signals regulating mesothelial cell transdifferentiaton and have used this atlas to examine *in vitro* models of mesothelioma.

## Supplementary material

10.1183/13993003.00143-2023.Supp1 **Please note:** supplementary material is not edited by the Editorial Office, and is uploaded as it has been supplied by the author.
Supplementary material ERJ-00143-2023.Supplement

## Shareable PDF

10.1183/13993003.00143-2023.Shareable1 This one-page PDF can be shared freely online.
Shareable PDF ERJ-00143-2023.Shareable
