# Supplementary material for: Single-cell transcriptomic analysis of human pleura reveals stromal heterogeneity and informs in vitro models of mesothelioma
Source: Eur Respir J. 2024 Jan 25;63(1):2300143. doi: 10.1183/13993003.00143-2023 (PMC10809128; doi:10.1183/13993003.00143-2023)
Supplement: Supplementary file 1 [file ERJ-00143-2023.Supplement.pdf]

FIGURE S1

A

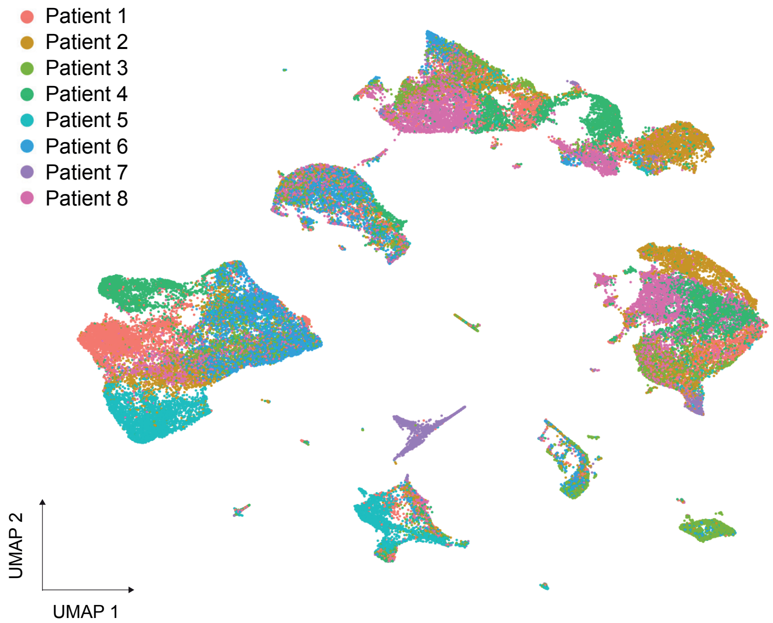

B

	Mesothelial	Fibroblast	Endothelial	Smooth Muscle	Neutrophils	T cells	B cells	Pericytes	Mast cells	Dendritic cells
Patient 1	2.00	2.90	5.97	0.61	0.58	0.23	0.04	0.08	0.05	0.02
Patient 2	4.53	5.07	3.09	0.65	0.40	0.39	0.03	0.33	0.04	0.03
Patient 3	1.91	3.00	2.81	0.93	0.36	1.33	1.82	0.33	0.04	0.08
Patient 4	4.54	4.85	3.53	0.46	0.29	0.03	0.03	0.35	0.05	0.01
Patient 5	0.37	0.57	5.27	0.13	3.07	0.08	0.04	0.14	0.20	0.19
Patient 6	0.00	1.63	6.93	2.71	0.28	1.22	0.30	0.48	0.04	0.02
Patient 7	0.66	0.35	0.00	0.00	0.05	2.35	0.00	0.00	0.03	0.02
Patient 8	6.10	7.79	2.57	1.57	0.47	0.13	0.03	0.39	0.08	0.00

Figure S1 scRNA-seq atlas of human parietal pleura by donor
A. Uniform manifold approximation and projection (UMAP) of 63,748 pleural cells from 8 donors are colour-coded by donor.
B. Table of relative abundance by cell type and donor. Values are percentages of the 63,748 pleural cells presented as a percentage of the whole.

FIGURE S2

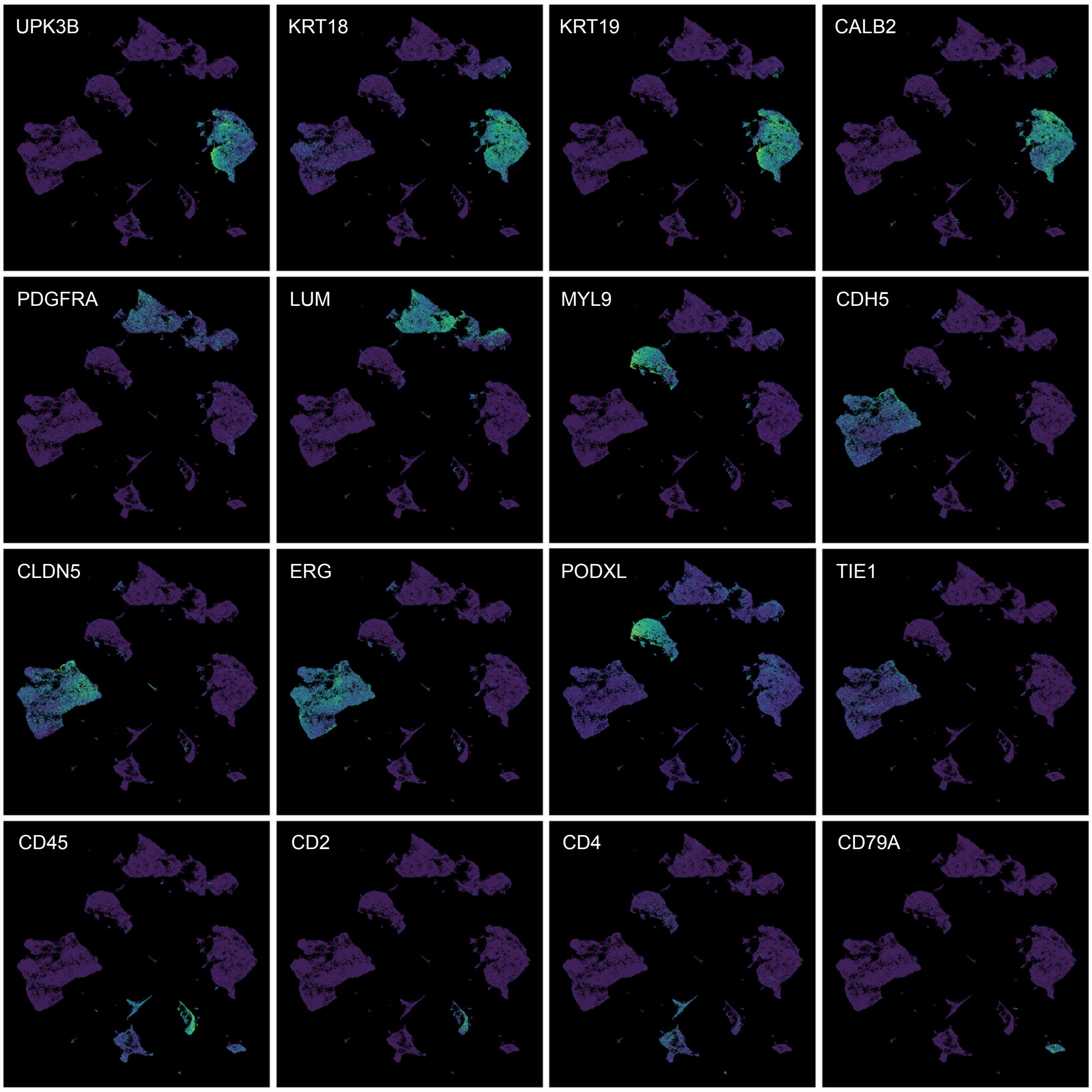
UPK3B
KRT18
KRT19
CALB2
PDGFRA
LUM
MYL9
CDH5
CLDN5
ERG
PODXL
TIE1
CD45
CD2
CD4
CD79A

Figure S2 Cell type identification

UMAP projection of canonical marker gene expression: mesothelial cells (UPK3B, KRT18, KRT19, CALB2); fibroblasts (PDGFRA, LUM); smooth muscle cells (MYL9); endothelial cells (CDH5, CLDN5, ERG, PODXL, TIE1); immune subtypes (CD45, CD2, CD4, CD79A).

A

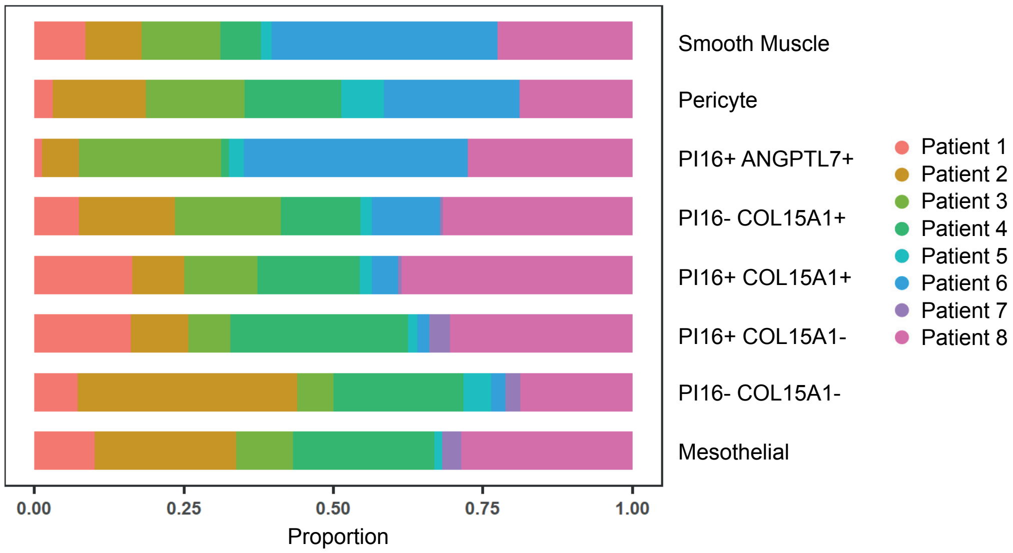

B

	Mesothelial	PI16- COL15A1-	PI16+ COL15A1-	PI16+ COL15A1+	PI16- COL15A1+	PI16+ ANGPTL7+	Pericyte	Smooth Muscle
Patient 1	3.55	1.03	0.92	2.21	1.09	0.00	0.11	1.10
Patient 2	8.34	5.20	0.55	1.17	2.34	0.02	0.57	1.22
Patient 3	3.32	0.85	0.40	1.64	2.60	0.06	0.60	1.70
Patient 4	8.35	3.09	1.69	2.30	1.94	0.00	0.59	0.89
Patient 5	0.45	0.64	0.09	0.27	0.27	0.01	0.26	0.22
Patient 6	0.02	0.35	0.12	0.59	1.66	0.09	0.83	4.91
Patient 7	1.11	0.35	0.19	0.08	0.08	0.00	0.00	0.00
Patient 8	10.09	2.65	1.74	5.20	4.63	0.07	0.69	2.92

Figure S3 Patient contribution to each cell type group

A. Relative abundance of stromal cell type and donor expressed graphically. Values are proportion of the 32,750 stromal cells.

B. Relative abundance by cell type and donor. Values are proportion of the 32,750 stromal cells presented as a percentage of the whole.

FIGURE S4

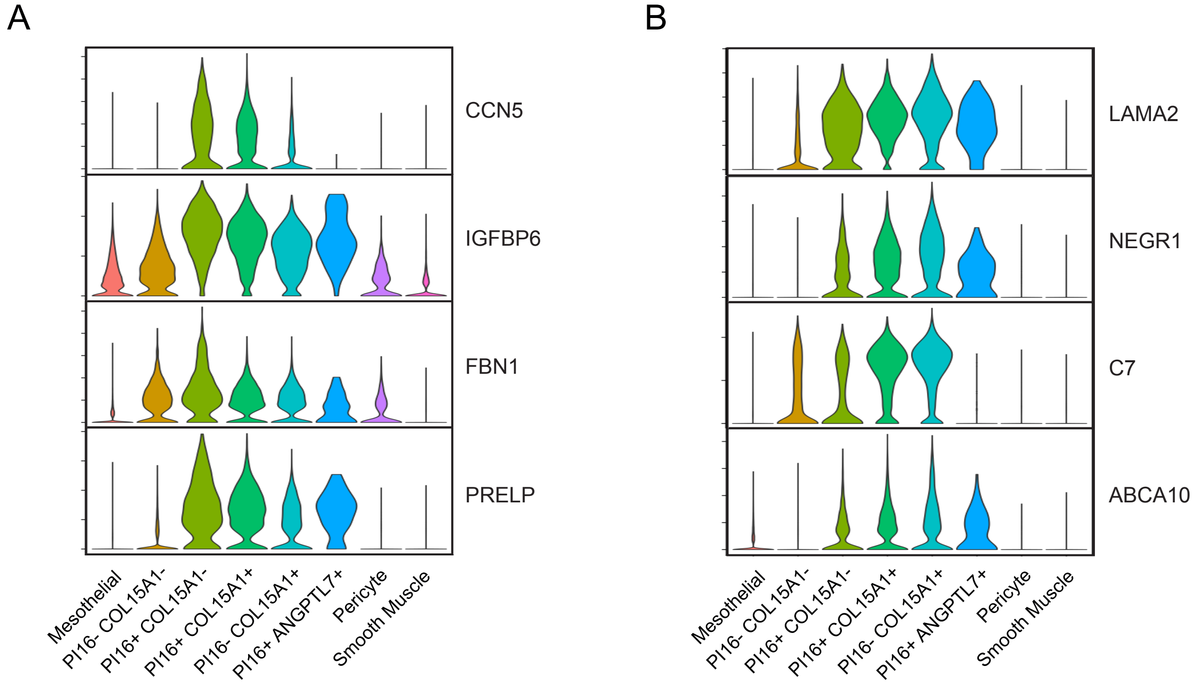
A
CCN5
IGFBP6
FBN1
PRELP
Mesothelial
PI16- COL15A1-
PI16+ COL15A1-
PI16+ COL15A1+
PI16- COL15A1+
PI16+ ANGPTL7+
Pericyte
Smooth Muscle
B
LAMA2
NEGR1
C7
ABCA10
Mesothelial
PI16- COL15A1-
PI16+ COL15A1-
PI16+ COL15A1+
PI16- COL15A1+
PI16+ ANGPTL7+
Pericyte
Smooth Muscle

Figure S4 Distribution of universal fibroblast markers

A. Violin plot of *PI16*+ *COL15A1*- fibroblast marker gene expression.
B. Violin plot of *PI16*- *COL15A1*+ fibroblast marker gene expression.

FIGURE S5

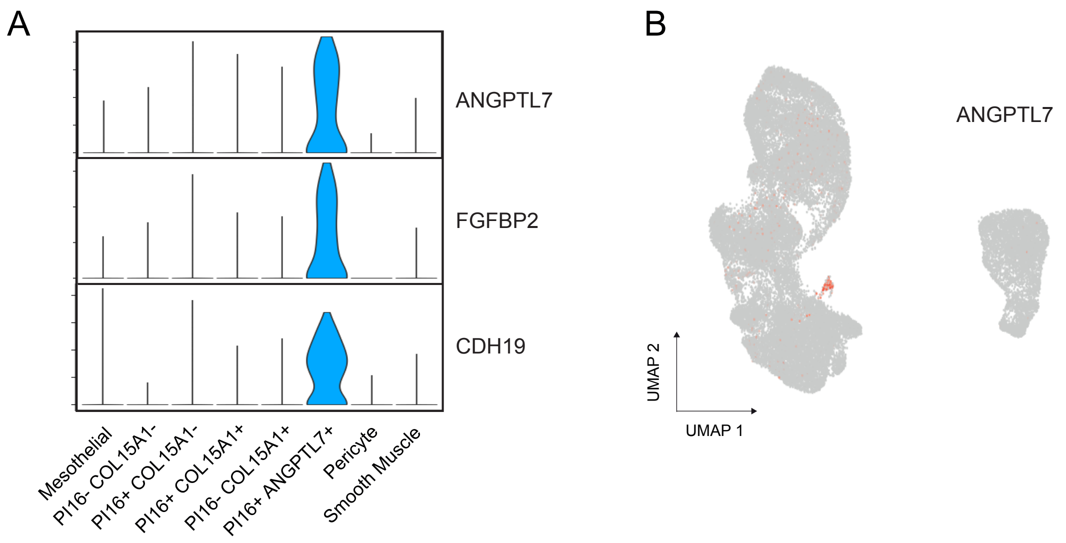
A
ANGPTL7
FGFBP2
CDH19
Mesothelial
PI16- COL15A1-
PI16+ COL15A1-
PI16+ COL15A1+
PI16- COL15A1+
PI16+ ANGPTL7+
Pericyte
Smooth Muscle
B
ANGPTL7
UMAP 2
UMAP 1

Figure S5 Additional ANGPTL7+ fibroblast marker genes
A. Violin plot of gene expression in ANGPTL7 fibroblasts. Note this population also expresses CDH19 and FGFBP2.
B. UMAP illustrating ANGPTL7+ cells.

FIGURE S6

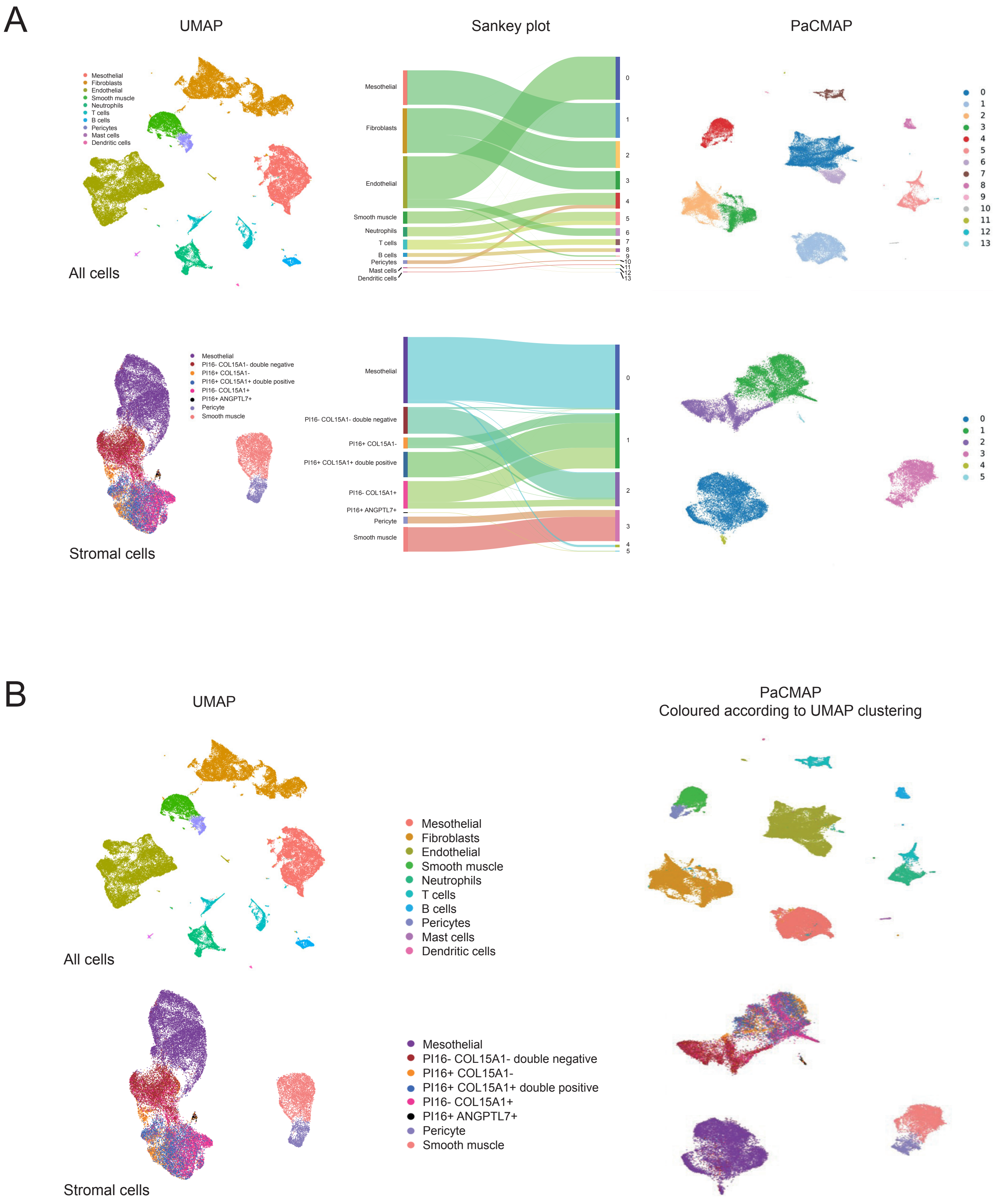
A
UMAP
Sankey plot
PaCMAP
Mesothelial
Fibroblasts
Endothelial
Smooth muscle
Neutrophils
T cells
B cells
Pericytes
Mast cells
Dendritic cells
All cells
0
1
2
3
4
5
6
7
8
9
10
11
12
13
Mesothelial
PI16- COL15A1- double negative
PI16+ COL15A1-
PI16+ COL15A1+ double positive
PI16- COL15A1+
PI16+ ANGPTL7+
Pericyte
Smooth muscle
Stromal cells
0
1
2
3
4
5
B
UMAP
PaCMAP
Coloured according to UMAP clustering
Mesothelial
Fibroblasts
Endothelial
Smooth muscle
Neutrophils
T cells
B cells
Pericytes
Mast cells
Dendritic cells
All cells
Mesothelial
PI16- COL15A1- double negative
PI16+ COL15A1-
PI16+ COL15A1+ double positive
PI16- COL15A1+
PI16+ ANGPTL7+
Pericyte
Smooth muscle
Stromal cells

Figure S6 PaCMAP dimensionality reduction and clustering
A. Left: UMAP dimensionality reduction and clustering of pleural and stromal pleural cells. Right: PaCMAP dimensionality reduction and new clustering of pleural and stromal pleural cells. Middle: Sankey plot detailing the initial UMAP clustering classification for each cell and the new clustering classification using PaCMAP.
B. Left: UMAP dimensionality reduction and clustering of pleural and stromal pleural cells. Right: PaCMAP dimensionality reduction coloured by the initial UMAP clustering classification for each cell.

FIGURE S7

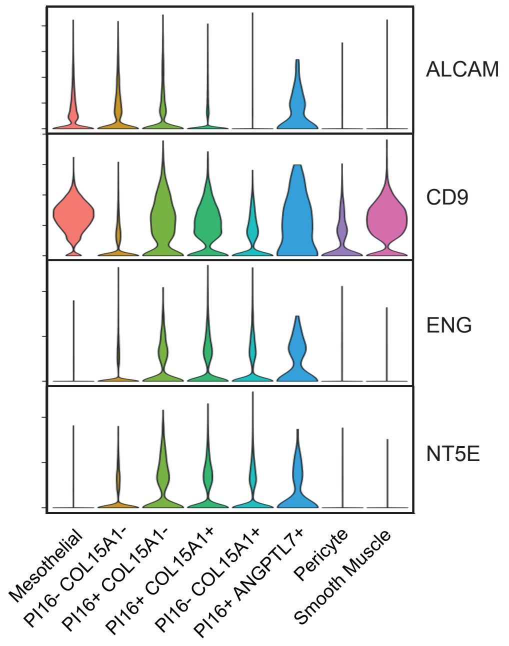
ALCAM
CD9
ENG
NT5E
Mesothelial
PI16- COL15A1-
PI16+ COL15A1-
PI16+ COL15A1+
PI16- COL15A1+
PI16+ ANGPTL7+
Pericyte
Smooth Muscle

Figure S7 Stemness gene expression
Violin plot of additional stemness-associated gene expression.

FIGURE S8

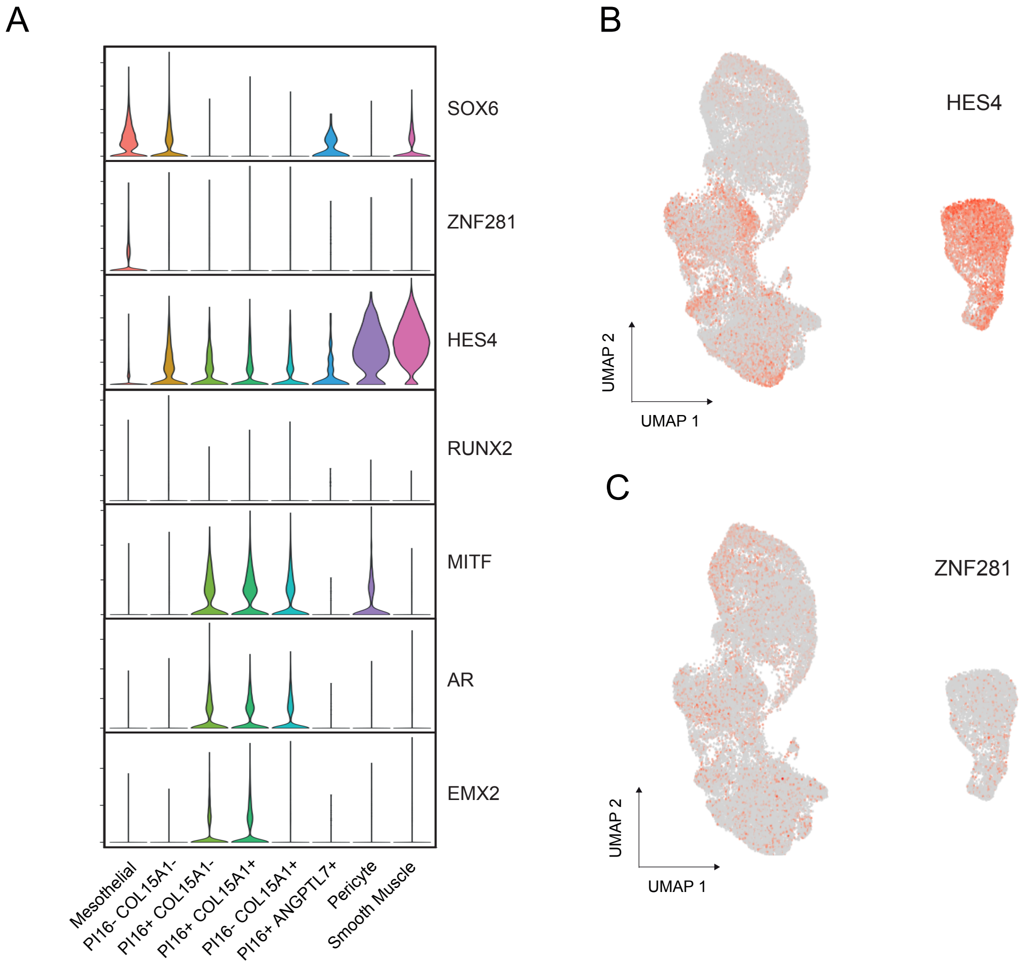
A
SOX6
ZNF281
HES4
RUNX2
MITF
AR
EMX2
Mesothelial
PI16- COL15A1-
PI16+ COL15A1-
PI16+ COL15A1+
PI16- COL15A1+
PI16+ ANGPTL7+
Pericyte
Smooth Muscle
B
HES4
UMAP 2
UMAP 1
C
ZNF281
UMAP 2
UMAP 1

Figure S8 Transcription factor expression in pleural fibroblasts

A. Violin plot of selected transcription factor expression in pleural stromal cell populations whose gene sets were observed active in some fibroblast populations in Figure 2C.

B. UMAP illustrating HES4+ cells.

C. UMAP illustrating ZNF281+ cells.

FIGURE S9

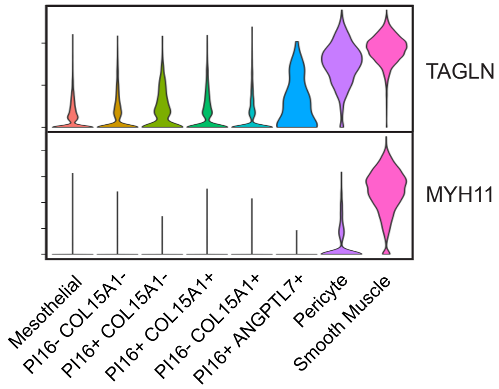
TAGLN
MYH11
Mesothelial
PI16- COL15A1-
PI16+ COL15A1-
PI16+ COL15A1+
PI16- COL15A1+
PI16+ ANGPTL7+
Pericyte
Smooth Muscle

Figure S9 Further myofibroblast markers
Violin plot of myofibroblast marker gene (TAGLN, MYH1) expression.

FIGURE S10

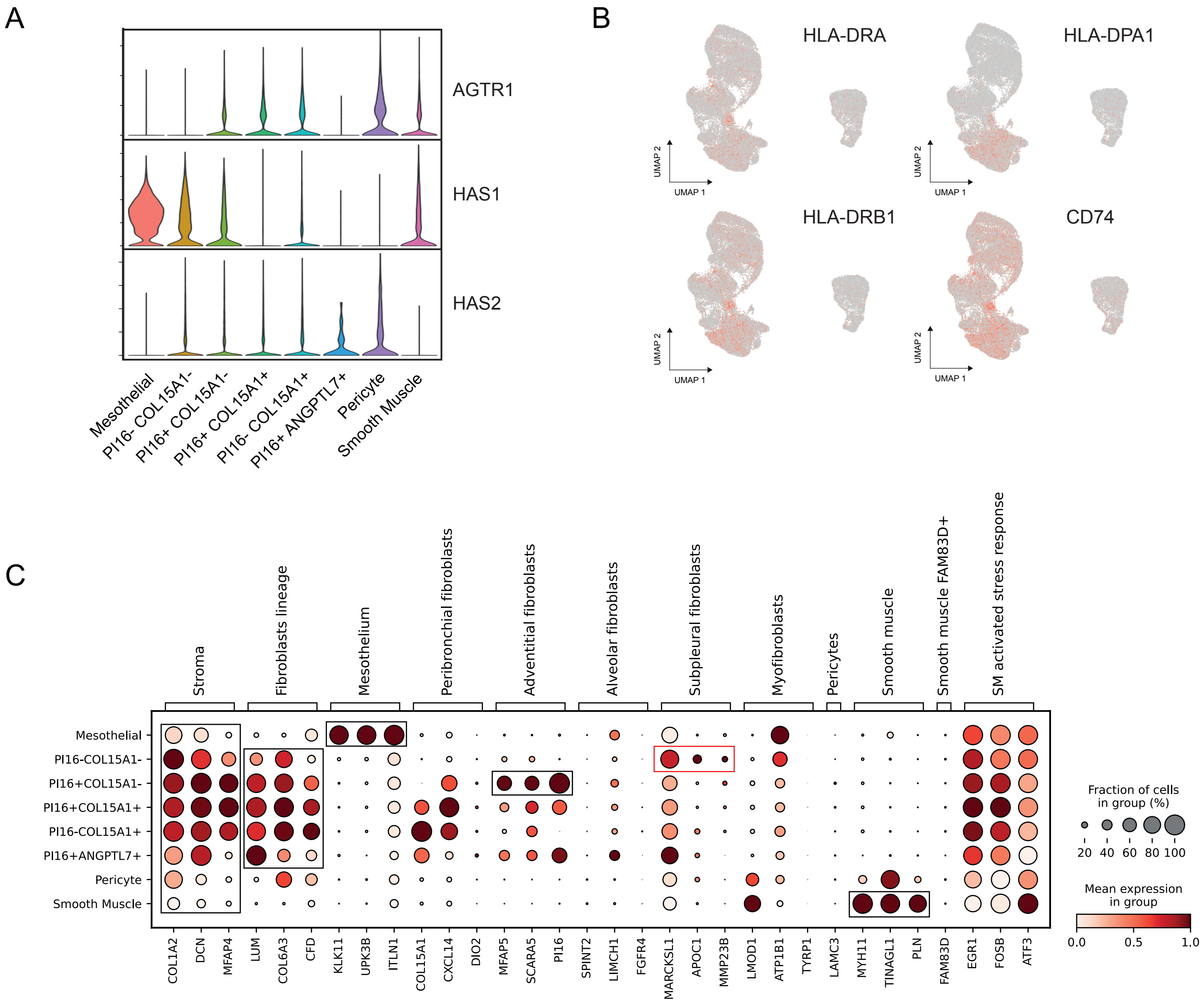
A
AGTR1
HAS1
HAS2
Mesothelial
PI16- COL15A1-
PI16+ COL15A1-
PI16+ COL15A1+
PI16- COL15A1+
PI16+ ANGPTL7+
Pericyte
Smooth Muscle
B
HLA-DRA
HLA-DPA1
HLA-DRB1
CD74
UMAP 1
UMAP 2
C
Stroma
Fibroblasts lineage
Mesothelium
Peribronchial fibroblasts
Adventitial fibroblasts
Alveolar fibroblasts
Subpleural fibroblasts
Myofibroblasts
Pericytes
Smooth muscle
Smooth muscle FAM83D+
SM activated stress response
Mesothelial
PI16-COL15A1-
PI16+COL15A1-
PI16+COL15A1+
PI16-COL15A1+
PI16+ANGPTL7+
Pericyte
Smooth Muscle
COL1A2
DCN
MFAP4
LUM
COL6A3
CFD
KLK11
UPK3B
ITLN1
COL15A1
CXCL14
DIO2
MFAP5
SCARA5
PI16
SPINT2
LIMCH1
FGFR4
MARCKSL1
APOC1
MMP23B
LMOD1
ATP1B1
TYRP1
LAMC3
MYH11
TINAGL1
PLN
FAM83D
EGR1
FOSB
ATF3
Fraction of cells in group (%)
20 40 60 80 100
Mean expression in group
0.0 0.5 1.0

Figure S10 Further cell markers
A. Violin plot of selected iCAF marker gene expression (AGTR1, HAS1, HAS2).
B. UMAP visualisation HLA-DRA, HLA-DPA1, HLA-DRB1, CD74
C. Cell type proportions following the method and marker genes of Sikkema et al[34]. Expression normalised so the maximum group expression of cells within the compartment for each marker was set to 1. Black boxes correspond to cell types identified by Sikkema et al. Red box indicates similarity to subpleural fibroblast subtype.

FIGURE S11

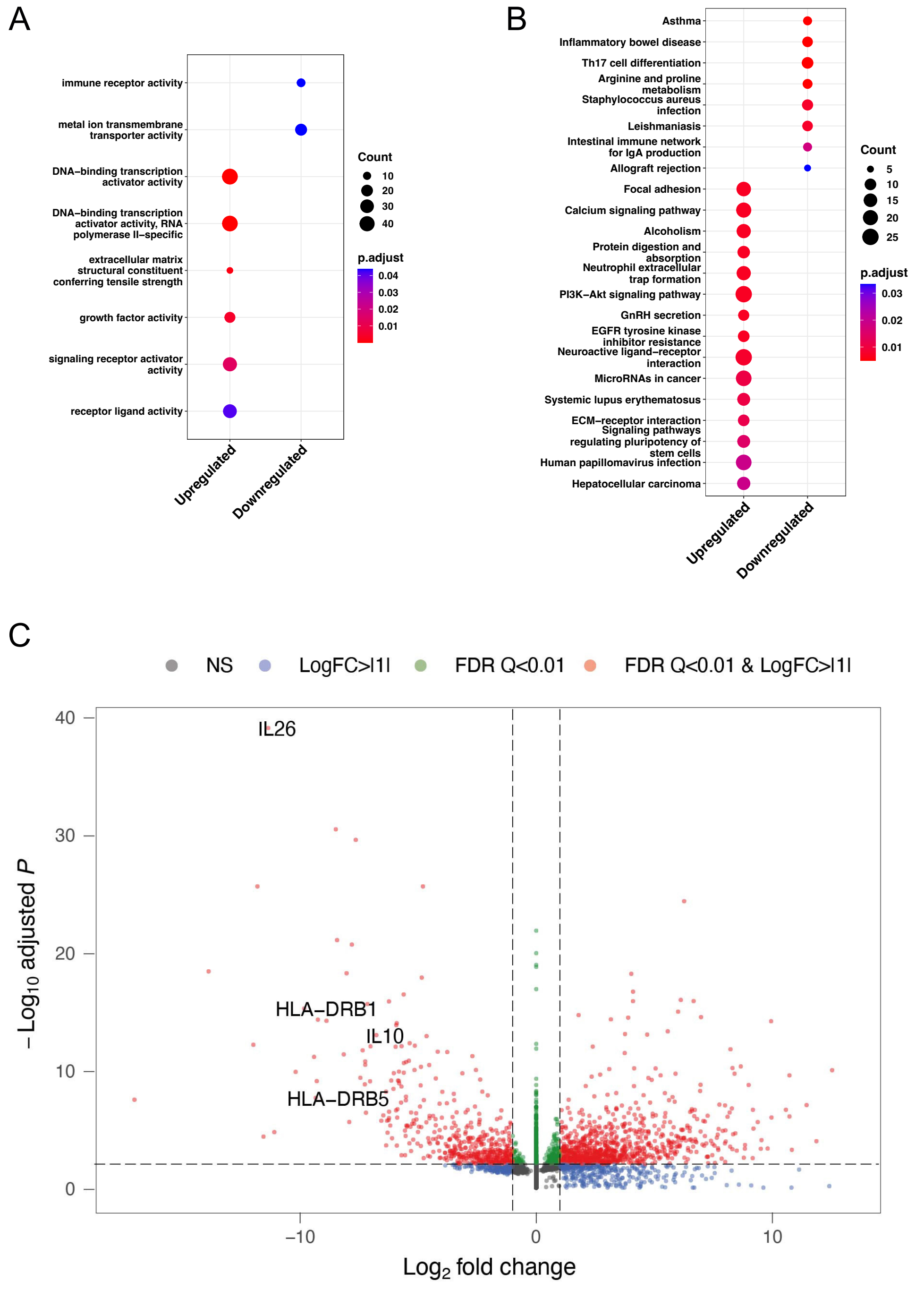

A
immune receptor activity
metal ion transmembrane transporter activity
DNA–binding transcription activator activity
DNA–binding transcription activator activity, RNA polymerase II–specific
extracellular matrix structural constituent conferring tensile strength
growth factor activity
signaling receptor activator activity
receptor ligand activity
Upregulated
Downregulated
Count
10
20
30
40
p.adjust
0.04
0.03
0.02
0.01
B
Asthma
Inflammatory bowel disease
Th17 cell differentiation
Arginine and proline metabolism
Staphylococcus aureus infection
Leishmaniasis
Intestinal immune network for IgA production
Allograft rejection
Focal adhesion
Calcium signaling pathway
Alcoholism
Protein digestion and absorption
Neutrophil extracellular trap formation
PI3K–Akt signaling pathway
GnRH secretion
EGFR tyrosine kinase inhibitor resistance
Neuroactive ligand–receptor interaction
MicroRNAs in cancer
Systemic lupus erythematosus
ECM–receptor interaction
Signaling pathways regulating pluripotency of stem cells
Human papillomavirus infection
Hepatocellular carcinoma
Upregulated
Downregulated
Count
5
10
15
20
25
p.adjust
0.03
0.02
0.01
C
NS
LogFC>|1|
FDR Q<0.01
FDR Q<0.01 & LogFC>|1|
IL26
HLA–DRB1
IL10
HLA–DRB5
40
30
20
10
0
–10
0
10
– Log10 adjusted P
Log2 fold change

Figure S11 Bulk RNA-seq analysis of healthy donor and mesothelioma cell cultures
A. Gene Ontology (GO) Term analysis of mesothelioma cell bulk RNA-seq relative to bulk RNA-seq of healthy mesothelial cell cultures (5-6 days post-isolation).
B. Similarly, KEGG pathway analysis of mesothelioma cell bulk RNA-seq relative to bulk RNA-seq of healthy mesothelial cell cultures (5-6 days post-isolation).
C. Volcano plot of mesothelioma cell bulkRNA-seq relative to bulk RNA-seq of healthy mesothelial cell cultures (5-6 days post-isolation).
