# Supplementary material for: Single-cell transcriptomic analysis of human pleura reveals stromal heterogeneity and informs in vitro models of mesothelioma
Source: Eur Respir J. 2024 Jan 25;63(1):2300143. doi: 10.1183/13993003.00143-2023 (PMC10809128; doi:10.1183/13993003.00143-2023)
Supplement: Supplementary file 2 [file ERJ-00143-2023.Shareable.pdf]

EUROPEAN RESPIRATORY JOURNAL
ORIGINAL RESEARCH ARTICLE
J. OBACZ ET AL.

SHAREABLE PDF

Single-cell transcriptomic analysis of human pleura reveals stromal heterogeneity and informs *in vitro* models of mesothelioma

Joanna Obacz [1,2,8], Jose Antonio Valer [1,2,8], Reshma Nibhani [2], Taylor S. Adams [3], Jonas C. Schupp [4], Niki Veale[1,2], Amanah Lewis-Wade[1,2], Jasper Flint[3], John Hogan[5], Giuseppe Aresu[5], Aman S. Coonar[5], Adam Peryt[5], Giulia Biffi[6], Naftali Kaminski [3], Hayley Francies[7], Doris M. Rassl[5], Mathew J. Garnett[7,9], Robert C. Rintoul [5,6,9] and Stefan J. Marciniak [1,2,5,9]

[1]Cambridge Institute for Medical Research (CIMR), University of Cambridge, Cambridge, UK. [2]Division of Respiratory Medicine, Department of Medicine, University of Cambridge, Cambridge, UK. [3]Section of Pulmonary, Critical Care, and Sleep Medicine, Yale School of Medicine, New Haven, CT, USA. [4]Department of Respiratory Medicine, Hannover Medical School, German Center for Lung Research (DZL), Hannover, Germany. [5]Royal Papworth Hospital NHS Foundation Trust, Cambridge, UK. [6]Cancer Research UK Cambridge Institute, University of Cambridge, Cambridge, UK. [7]Wellcome Sanger Institute, Wellcome Genome Campus, Hinxton, UK. [8]Joint first authors. [9]Joint senior authors.

Corresponding author: Stefan J. Marciniak (sjm20@cam.ac.uk)

Check for updates

Shareable abstract (@ERSpublications)
The first single-cell RNA-seq atlas of human parietal pleura is now accessible *via* a free web-based data-mining tool, MesothelialCellAtlas.com, and illuminates aspects of benign and malignant pleural biology https://bit.ly/3u3eDu8

Cite this article as: Obacz J, Valer JA, Nibhani R, *et al*. Single-cell transcriptomic analysis of human pleura reveals stromal heterogeneity and informs *in vitro* models of mesothelioma. *Eur Respir J* 2024; 63: 2300143 [DOI: 10.1183/13993003.00143-2023].

This extracted version can be shared freely online.

Copyright ©The authors 2024.

This version is distributed under the terms of the Creative Commons Attribution Licence 4.0.

Received: 26 Jan 2023
Accepted: 30 Oct 2023

Abstract

The pleural lining of the thorax regulates local immunity, inflammation and repair. A variety of conditions, both benign and malignant, including pleural mesothelioma, can affect this tissue. A lack of knowledge concerning the mesothelial and stromal cells comprising the pleura has hampered the development of targeted therapies. Here, we present the first comprehensive single-cell transcriptomic atlas of the human parietal pleura and demonstrate its utility in elucidating pleural biology. We confirm the presence of known universal fibroblasts and describe novel, potentially pleural-specific, fibroblast subtypes. We also present transcriptomic characterisation of multiple *in vitro* models of benign and malignant mesothelial cells, and characterise these through comparison with *in vivo* transcriptomic data. While bulk pleural transcriptomes have been reported previously, this is the first study to provide resolution at the single-cell level. We expect our pleural cell atlas will prove invaluable to those studying pleural biology and disease. It has already enabled us to shed light on the transdifferentiation of mesothelial cells, allowing us to develop a simple method for prolonging mesothelial cell differentiation *in vitro*.
